# Importance of Social Hierarchy in Morphometry, and Socio-Sexual and Reproductive Behaviors in Dorper Sheep in Northern Mexico

**DOI:** 10.3390/ani16060994

**Published:** 2026-03-23

**Authors:** Silvestre Moreno-Avalos, Miguel Angel Gaytan-Aguilera, Aracely Zuñiga-Serrano, Francisco Gerardo Véliz-Romero, Edgar Díaz-Rojas, Rafael Rodríguez-Martínez, Viridiana Contreras-Villarreal, Martín Alfredo Legarreta-González, Cayetano Navarrete-Molina, Francisco Gerardo Véliz-Deras

**Affiliations:** 1 Regional Division of Animal Science, Antonio Narro Agrarian Autonomous University, Laguna Unit, Torreon, Coahuila 27054, Mexico; 2 Technological University of the Tarahumara, Guachochi, Chihuahua 33180, Mexico

**Keywords:** high social rank, low social rank, anestrous, male effect, reproductive efficiency

## Abstract

One of the most important aspects of animal production is the reproductive behavior, where a widely used strategy in small ruminants is the “male effect”. However, the response to this effect can vary depending on several factors, including the social rank (SR) of both sexes. The objective of this study was to evaluate how SR influences morphometric and socio-sexual variables in Dorper sheep in Northern Mexico. Through behavioral tests, 33 rams and 59 ewes were divided into two groups taking into account their SR. Rams included high SR (HSR; n = 14) and low SR (LSR; n = 19), while ewes included HSR (n = 23) and LSR (n = 36). Morphometric response variables included age, live weight, body condition, thoracic circumference, wither height, presence of horns, and testicular circumference, whereas reproductive variables included estrus rate, ovulation rate, corpus luteum, left ovary, right ovary, pregnancy rate, and embryos’ number. The results showed no differences (*p* > 0.05) for the morphometric variables considered, whilst, regarding the reproductive variables, the LSR rams × HSR ewes group showed the highest number of embryos (*p* < 0.05), and the corpus luteum number was higher in the HSR rams × HSR ewes group (*p* < 0.05). However, no significant differences were found for the remaining variables (*p* > 0.05). Improving productive and reproductive performance can contribute to increased income for sheep farmers.

## Simple Summary

Improving the productive and reproductive performance of sheep flocks presents a significant challenge. Implementing strategies (e.g., the male effect × social rank) can contribute to this challenge. The objective of this study was to evaluate how social rank influences morphometric and socio-sexual variables in Dorper sheep in Northern Mexico. Through behavioral tests, 33 rams and 59 ewes were divided into two groups, taking into account their social rank (i.e., high social rank and low social rank). The results showed no significant differences (*p* > 0.05) for the morphometric variables considered. Meanwhile, regarding the reproductive variables, the combination of low-social-rank rams × high-social-rank ewes resulted in the highest number of embryos (*p* < 0.05), while the corpus luteum number was higher for the combination of high-social-rank rams × high-social-rank ewes (*p* < 0.05). However, no significant differences were observed for the remaining variables (*p* > 0.05). These results contribute to the ongoing effort to improve reproductive performance in sheep, enhancing the sustainability of the activity, with the potential to increase the income of families who depend on sheep production.

## 1. Introduction

Reproductive performance is one of the most important and limiting aspects in animal production, considering that it affects, in a crucial way, the profitability of livestock farms [1,2,3,4,5]. In this regard, seasonality, which has been related to thre photoperiod, represents one of the main factors that affects the productive performance, particularly in small ruminants, limiting the reproductive efficiency and rate regardless of the animal’s sex [6,7,8,9]. For this reason, producers and researchers are forced to use diverse strategies, in order to achieve a higher productivity and reproductive efficiency on farms. In this sense, a strategy used in sheep is the phenomenon known as the “male effect”, which refers to the stimulation throughout chemical and auditive signals, effected by males over females in shallow anestrus or close to a breeding season, and which culminates in ovulation and the onset of estrus in females [10,11,12,13,14,15].

However, the females’ response to the rams’ effect can vary depending on other factors, such as the male’s sexual behavior, race, and age and both animals’ social rank [3,6,7,10,12]. In regard to small ruminants (i.e., sheep), the social rank determines the access to the available resources; for example, it has been reported that high-social-rank (HSR) males display a better sexual behavior than the low social rank (LSR), and that they can also inhibit their sexual behavior [1,16]. Hence, the sheep’s reproductive success increases in connection with the individual’s social rank; for example, in sheep, HSR females ovulate and get pregnant before LSR ones, because HSR sheep monopolize the HSR male’s interaction [1,17,18]. In the same way, LSR females usually have lower energy reserves, which limits the expression of behaviors like the search for males or the sexual receptivity [17,19]; and, likewise, HSR male sheep can inhibit the LSR reproductive response [16,20]. However, despite the fact that HSR and LSR males have the ability to induce the females’ sexual activity [1,18], it has been registered that HSR males stimulate, with a higher efficiency, the females’ reproductive activity [1,6,9,18]. Furthermore, LSR males exhibit more rejections and fewer mounts than HSR males [21].

In addition, it has been reported that the female’s body condition and diet can also significatively affect their response to the rams’ effect [6], inducing HSR animals to show higher weight and energy reserves than the LSR ones [18,21]. Likewise, HSR males have a higher testicular capacity than LSR males [1,18,21]. Nevertheless, in the case of sheep, it has been documented that, animals with a different social rank do not always show morphometric differences (i.e., horns and thoracic capacity) between them [21]. Taking into account the previous findings, we present that the social rank in sheep flocks in northern Mexico modulates reproductive and behavioral responses to the male effect. Therefore, the objective of this investigation was to evaluate the social rank’s influences over morphometric variables and the socio-sexual response to the male effect, in northern Mexico. From a sustainability perspective, improving the reproductive efficiency of sheep can contribute to an increase in the family’s income, which aligns with the Sustainable Development Goals.

## 2. Materials and Methods

### 2.1. General

All experimental procedures and animal management used in this investigation complied with international [22] and national [23] standards for ethical use, care, and welfare of animals in research. Additionally, this investigation had institutional approval, with reference number UAAAN-UL-18-3059.

### 2.2. Location Description and Environmental Conditions

This study was conducted in the north of Mexico, in the region named Comarca Lagunera (25° 37′ N, 103° 16′ W, 1113 masl). The region is classified as an arid ecosystem, comprising 86% alluvial plains, with dry and warm weather, and with an average annual temperature of 22.2 °C. Recording an average maximum temperature of 41.2 °C for spring and an average minimum temperature of −5.8 °C for winter. The region has an average of 173.1 mm annual rainfall, with a relative humidity that oscillates between 12% and 61%, and with light variations that range from 13 h 41 min during the spring solstice (June) to 10 h 19 min during the winter solstice (December) [24,25].

### 2.3. Experimental Animals

**Figure 1:**
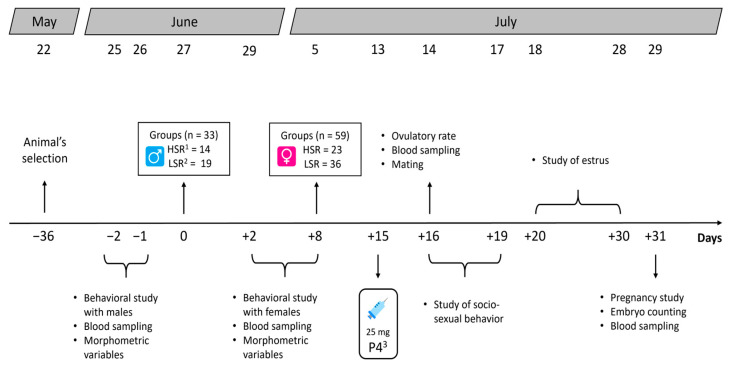
Timeline of experimental procedures during the study. The evaluation of the social rank in Dorper sheep was carried out during the non-breeding season (May) in northern Mexico (25° N).

A total of 33 rams of 35 available in the flock were selected and divided into two different groups, based on their social rank (i.e., high social rank (HSR), n = 14; and low social rank (LSR), n = 19). Regarding the females, a random sample of 59 Dorper ewes was selected based on the established experimental design and divided into two groups based on their social rank (i.e., HSR, n = 23; and LSR, n = 36). The flock’s management behooves to an intensive production system, with a feeding system consisting of providing them food twice a day, including 300 g of alfalfa hay and commercial concentrate (15% crude protein) per head per day, as well as free access to water and minerals. Thirty-six days before the start of the experiment, the sheep’s reproductive state was confirmed by ultrasound (Chison ECO5 Doppler, Wuxi, Jiangsu, China), using a rectal transducer (7.5 MHz, Doppler Color, Chison^®^, Wuxi, Jiangsu, China). At the time of the operation, the transducer was lubricated and introduced into the sheep’s anus, to detect and exclude pregnant females from this study. The investigation took place during the months of May and July 2024, which involved the transition period to mating (May–June) and the reproductive season (June–July) (Figure 1).

### 2.4. Social Rank Determination (Behavioral Tests)

#### 2.4.1. Males

In order to determine the males’ social rank, a behavioral test was performed. For this reason, ram-to-ram competency studies were carried out, with rams’ pairs being exposed to females in estrus inside 2.5 m × 2.5 m pens. Each behavioral evaluation involved exposing each sheep to a pair of males for 30 min and assessing their sexual behavior. Subsequently, new pairs of rams were formed until each male competed with the rest of the male group. The male’s social rank was determined through the success index (SI), described previously for sheep by Véliz-Deras et al. [21], taking into account the following male-to-male behaviors: bumps, threats, shoves, chases, escapes, and evasions. In addition, when a male showed dominant behavior toward another male who declined an interaction (the subordinate male), it was considered a male-to-female interaction. The socio-sexual behaviors registered for the male-to-female interaction included bumps, anogenital sniffing, flehmen, foreleg kicks, bare, self-marking, blockade, mount, bare mount, incomplete mount and mount with intromission. The reproductive behavioral observations were recorded by ten people previously trained and qualified for the task. In order to calculate the SI, the number of events won were divided between the total number of events, and subsequently, employing the aforementioned index, the males were divided in the following social ranks: low (LSR; SI = 0 to 0.5) and high (HSR; SI = 0.51 to 1.0), which were an adaptation of the ranges employed by Véliz-Deras et al. [21].

#### 2.4.2. Females

To determine the females’ social rank, a study of competitive behavior over food was conducted. For this purpose, the 59 ewes were housed in a 20 m × 40 m pen, with a feeder located along the 20 m width of the pen’s side. We allowed the sheep access to the feeder from only one side of the feeder. In addition, the pen also included two 1 m × 0.5 m drinking troughs at the opposite end, as well as two mineral salt blocks. The behavior evaluations were conducted for one hour (08:00 h), over eight consecutive days at the morning mealtime. For this, the main interactions between sheep were observed: bumps, threats, shoves, chases, escapes, and head butts. All those interactions were documented, considering that, between two individuals, there is always a victim and an instigator involved, regardless of whether or not any physical contact ever occurred. With the aid of the data obtained from the agonistic interactions, the SI was calculated, and, utilizing the aforementioned methodology, the females were classified in two different social ranks: low (LSR; SI = 0 to 0.5) and high (HSR; SI = 0.51 to 1.0).

### 2.5. Socio-Sexual Behavioral Tests

Each group of male and female Dorper sheep (i.e., HSR and LSR) was housed in 20 m × 20 m pens under the same environmental and management conditions. Subsequently, both groups of rams and ewes were mixed in two 20 m × 40 m pens for two days in a randomly determined combination. We obtained the following combinations: For the first two days, HSR males (n = 14) were allocated with LSR females (n = 36) in the first pen, whereas, in the second pend, LSR rams (n = 19) were housed with HSR ewes (n = 23). Consequently, on days three and four, an exchange of males was performed on both pens, resulting in pen number one containing LSR males with LSR females, and pen number two housing HSR rams with HSR ewes. The sexual response variables evaluated were anogenital sniffing, flehmen, foreleg kicks, bare, self-marking, blockade, mount, bare mount, incomplete mount and mount with intromission. The study lasted four days, giving two days to each male group with each group of females. All the male-to-female socio-sexual conducts observed were documented individually by ten people per pen, previously trained and qualified for the task. In addition, females from both social ranks received an intramuscular dose of progesterone (25 mg) (Progestelas “E”^®^, Aranda, Jalisco, Mexico) 24 h before their exposure to males, with the aim to avoiding short estrous cycles [26].

### 2.6. Variables Evaluated

#### 2.6.1. Morphometric Variables

The morphometric response variables, considered in the investigation, included the animal’s age (years), live weight (kg), length (cm), wither height (cm), thoracic circumference (cm), scrotal circumference (cm; only males), horn presence (yes/no) and body condition (units). The body condition was recorded through palpation of the muscle and adipose mass in the space between the spinous and transverse processes of the lumbar vertebrae, with a range from 1 to 4 (where 1 = emaciated, and 4 = obese) [27,28].

#### 2.6.2. Reproductive Response

The reproductive response variables considered for the investigation were ovulation, pregnancy, embryos and corpus luteum number, and sizes of right and left ovaries (mm). These were measured in this study 24 h after the progesterone application. Estrus was recorded for 15 consecutive days, using non-toxic paint in the chest for males, and recording the marked sheep number. Ovulation, the number of embryos, and corpus luteum were assessed via rectal ultrasound, which consisted of counting the number of embryos inside the uterus, four days after the males’ entrance, after the end of the socio-sexual behavioral evaluation. On the same line, pregnancy was diagnosed 31 days after the males’ entry to the females’ pen. And, in the case of mating, the males were exchanged every 24 h, leaving two males for each female groups. All the determinations made with the help of an ultrasound were performed using a rectal lineal transducer (7.5 MHz, Doppler Color, Chison^®^, Wuxi, Jiangsu, China), with water-based lubricating gel.

### 2.7. Statistical Analysis

After verifying the data sphericity needed to perform the selected statistical tests, the reproductive and morphometric variables were evaluated through a Chi-squared test and Fisher’s exact test. In addition, we employed an ANOVA and *t*-Student test in order to determine the differences (*p* < 0.05) between the multiple treatments and groups; subsequently, Tukey comparisons were performed to verify the results. The socio-sexual behaviors were analyzed through a generalized linear mixed model (PROC MIXED), employing a Poisson distribution and a logarithmic link, with the aim of evaluating the effects of the type, movement, and treatment. The statistical program R was used to conduct the aforementioned tests.

## 3. Results

Table 1 presents the number of confrontations won and lost per animal, which were utilized in the determination of both males’ and females’ social rank used in the present investigation. Similarly, Table 2 shows the comparison between both social rank individuals (i.e., HSR and LSR males and females) with respect to the different morphometric variables considered in this investigation. This evidences the absence of statistical difference between both ranks’ means (*p* > 0.05) in the same sex for each variable.

Table 3 presents the results obtained for each socio-sexual interaction. In general, it shows that HSR rams (HSR-R) evidenced a better behavior towards LSR ewes (LSR-E). In addition, it was evidenced that HSR-R presented greater stability in some of the variables than the LSR-rams (LSR-R). In regard to the reproductive variables (i.e., estrus, corpus luteum, ovary size, pregnancy rate and embryo number), the results showed that the experimental group of LSR-R with HSR ewes (HSR-E) [LSR-R × HSR-E] presented the highest number of embryos; this variable differed from the rest of the experimental groups (*p* < 0.05). When considering the corpus luteum number, the experimental group HSR-R × HSR-E reported a superior result compared to the rest of the groups (*p* < 0.05). On the same line, when analyzing reproductive behavior between female groups (i.e., HSR-E × LSR-E), it was determined that, for the variables, as well as the number of embryos and the corpus luteum, HSR-E showed higher values than its counterpart (*p* < 0.05). However, for the rest of the variables considered, no differences were observed (*p* > 0.05) (Table 4).

## 4. Discussion

Taking into account the obtained results, it is possible to affirm that the starting hypothesis remains unrefuted, by proving that the social rank exerts a modulatory influence in sheep flocks’ reproductive and behavioral response to the male effect, in northern Mexico. In that sense, HSR-R showed a higher SI than LSR-R, with HSR-R achieving victory in competition events 68.8% of the time (Table 1). This outcome seems to evidence a competitive advantage from HSR-R in behaviors associated with dominance and reproductive success, a pattern analogous to the one observed in ewes in the same aspect. On the other hand, it was obtained that both sexes had a greater number of animals in the LSR group compared to the HSR group, a characteristic that has to be taken into account when conducting statistical analysis, as it may necessitate a modification in the experimental design based on the differences in the number of animals. In addition, it has been demonstrated that the SI in sheep competition tests is influenced by genetic, physiological, environmental and management factors. Certain variables—such as lineage quality, age, size, weight, and body condition stand out—are decisive in aspects such as strength, resistance and the animal’s capability [29]; however, the findings in this study imply that some of these variables might not have an influence on that aspect.

The obtained results suggest that no significant difference was found (*p* > 0.05) between male and female groups, in most of the morphometric variables. In that sense, Zuñiga-Garcia et al. [30] reported that morphometric variables such as liveweight and body condition registered higher values in HSR goat groups, whereas Pascual-Alonso et al. [31] identified a connection between social strategies and morphometric and behavioral traits. These results suggest the existence of other variables with the ability to influence the sheep’s social rank. In addition, the results obtained in this investigation indicate that HSR-R groups exhibit a greater activity than LSR-R groups, in various socio-sexual behaviors. Concurrently, when performing the sexual behavior test in rams and sheep, SIs of 0.69 and 0.88 were recorded, respectively. This was sufficient to consider the individuals part of the HSR group, given the previously mentioned results, declared according to the aforementioned modification of the values established by Álvarez et al. [17].

In this study, the morphologic variables were also compared, and the results indicate that there were no significant differences between HSR-R and LSR-R groups (Table 2). However, research has demonstrated that aspects such as age, breed, and other factors play a major role in the expression of desirable sexual characteristics. This variability can be attributed, among other factors, to artificial selection and other parenting methods. An example of this can be seen in intensive management systems, where producers used to select replacements without placing too much importance to the animal’s sexual behavior [32]. Despite that, Kenyon et al. [33] mention that, under adequate feeding, a higher number of offspring can be obtained, and that the live weight and body condition play a major role in that aspect, when concluding that higher-liveweight females had a higher probability of mating within the first 17 days, resulting in a greater number of mating instances. Furthermore, it was reported that the use of isolated males can increase the number of mated females, leading to an augmentation of the conception and pregnancy rates [33].

On the other hand, it is important to acknowledge that the sheep’s reproductive season in northern Mexico changes depending on the breed; however, most of breeds show a less seasonal reproductive behavior in the study zone, due to the hot and arid climate in the region, which allows them to reproduce multiple times in the year. In this regard, it has been reported that the Khatahdin, Dorper, Blackbelly, Romanov, and Pelibuey breeds exhibit low reproductive seasonality in northern Mexico [34,35,36]. In the same context, it is possible to point out that the results of the comparison of the experimental groups evidenced significative differences in all the treatments, for some of the reproductive variables taken into account (Table 3 and Table 4); nevertheless, despite the differences in the number of individuals within each group, it was demonstrated that this characteristic did not exert an influence in most of the morphometric variables.

Regarding the estrus response variable, the results showed that, despite the presence of a discrepancy of 17.3% between the highest and lowest percentages reported, the results were not statistically significant between groups (*p* > 0.05), where a difference of from two to four ewes in estrus was not enough to be considered statistically significant. This result was consistent with the data reported by Véliz-Deras et al. [9], who did not encounter any statistical difference between the estrus and reproductive responses, in sheep exposed to the males’ effect, for rams treated with vitamin E and selenium. In this sense, the results published by Cadena-Villegas et al. [37] demonstrate that the males’ effect did not influence the estrus response in wool sheep synchronized with two doses of prostaglandins, and, in a similar study, Véliz-Deras et al. [21] documented that the sheep’s sexual response to the rams’ presence was similar for all groups in the study, with the researchers concluding that both HSR-R and LSR-R are adequate to induce an estrus response. However, it is imperative to acknowledge that the previous result could be influenced by the natural variability between individuals; the biological, environmental, and experimental factors; and the number of animals in the groups.

In addition, it has been reported that HSR-R exhibit a more active and consummatory sexual behavior than LSR-R [28], while rams with a higher dominant sexual behavior induce a greater number of ewes to enter estrus, accelerating the reproductive response, which suggests that the observed differences may be attributable to a natural variability among social groups [11]. Furthermore, Martin et al. [38] reported that nutrition is one of the environmental factors with the greatest influence on sheep reproduction. Therefore, it is possible to conclude that ewes’ sexual response to the presence of a ram depends on different factors, including the intensity of the stimulus and the ewe’s own capacity to respond. However, it is important to consider the possibility that some ewes may not respond to the male’s effect, even when exposed to highly stimulating rams (seasonal breeds); and, conversely, there may also be ewes who will have a weak response to the stimulus (seasonal less breeds) [39].

On another note, it is important to point out that the results obtained in this study in relation to the estrous response coincide with those reported by González-Tavizón et al. [18], where more than 80% of ewes entered estrus under the influence of rams, regardless of their social rank. This efficiency can be associated with a reproductive mechanism that increases fertilization, even when environmental factors such as the photoperiod limit ovarian activity [8,40]. Meanwhile, regarding the ovulation rate, this study indicates a relationship between the number of days over which the females were exposed to the males and their response, a result that coincides with the data reported by Chemineau [41], given that more than 90% of the females in both groups entered estrus during the first 10 days after interaction with the males. On the same line, it has been demonstrated that sheep under the same environmental conditions (photoperiod) can contribute to the onset of estrus in non-ovulating females [42], which is consistent with the findings of Pellicer-Rubio et al. [43], who considered that the presence of rams can trigger a rapid endocrine response in anestrous ewes, involving an increase in gonadotropin-releasing hormone and luteinizing hormone pulse release frequency. This is a response that can culminate in a preovulatory luteinizing hormone surge throughout a period of 3 to 30 h, concluding in ovulation within 24 to 60 h after the ram’s introduction. These results concur with those obtained by Cedillo-Ramírez and Flores-Cabrera [1], who reported that exposing anestrous ewes to HSR-R (78.3%) and LSR-R (69.3%) does not affect the ovulation rate after the first few days of exposure. Similarly, Knights et al. [44] determined that the sole introduction of rams constitutes an efficacious method to increase luteinizing hormone pulses, follicular development, and ovulation in ewes; meanwhile, Perkins and Fitzgerald [45] concluded that exposing ewes to rams with high reproductive capacity resulted in a higher percentage of ovulating ewes (95%), in comparison with those exposed to rams with a dismissed reproductive capacity (78%).

The aforementioned results concerning the ovulation rate are of great importance, considering that, although the male effect may have a positive impact on females, this does not necessarily result in a significant increase in their ovulation rate; and even if it does increase the ovulatory rate, there is still a possibility that females may exhibit a normal ovulation cycle [46]. It is therefore important to emphasize that, in accordance with the data obtained from Cedillo-Ramírez and Flores-Cabrera [1], and contrary to the results reported by Perkins and Fitzgerald [45], the combinations that included LSR-E showed a non-significant 16.7% lower ovulation percentage (Table 4) in comparison with those with HSR-E. These are outcomes that may be attributable to the observation that the animals from both groups belong to a lower social rank.

Regarding the corpus luteum number, no differences were identified between the experimental groups; however, a comparison of HSR-E × LSR-E revealed that the HSR group showed different values (*p* < 0.05) when compared with the LSR group. This finding agrees with the data reported by Arellano-Lezama et al. [20], who documented that, although no significant difference was recorded between the groups, the introduction of rams to a sheep flock increased the luteinizing hormone levels, stimulating progesterone production and the formation of corpus luteum; furthermore, they registered, as a secondary effect, an improvement in the reproductive efficiency due to an increase in the number and size of corpus luteum [5], since larger follicles indicate a better reproductive function and higher pregnancy rates [13,47].

However, it is important to point out that there is no consensus regarding the number and diameter of follicles in the presence of the male effect [14,48]. Hence, it is imperative to consider that these findings might suggest that the male effect induces ovulation, and that factors such as the social rank may exert a significant role in this process. Therefore, it is reasonable to assume that ovarian development and ovulation are processes regulated by factors such as the nutritional status, genetics, and management [49].

In the case of the pregnancy rate, it was determined that the most favorable outcomes were obtained when exposing an HSR group to an LSR one, regardless of the gender of the animals. In this sense, Stellflug et al. [50] posit that rams with a high sexual performance impregnated more ewes (499) than those with a lower one (258), and they achieved a higher prolificacy rate (1.52 vs. 1.38). Meanwhile, research such as the study conducted by Fowler and Langford [51] has demonstrated that pregnancy rates are linked to the rams’ social rank and fertility. Despite the fact that the pregnancy rates remained similar for all groups in this study, it is important to consider that the ram’s age plays a significant role. As has been demonstrated in the extant literature, experienced males induce a better reproductive response in comparison with young or inexperienced males, leading to the reporting of differences in the quantity and quality of stimuli, which is related to a smaller number of copulations and, consequently, to a lower pregnancy rate [52,53]. In this sense, the results obtained in this study are consistent with those reported by Contreras-Solis et al. [54], where ewes, treated with cloprostenol and exposed to the male effect in the early luteal phase, showed no differences in regard to the pregnancy rate in inseminated ewes. However, the findings of this study demonstrate a 18.2% discrepancy deemed as non-significant, which may be related to the differences in the group size. Nonetheless, Garza-Brenner et al. [55] found that the social rank does influence the reproductive outcome, as HSR males reported a higher number of twin births (52.9%) when compared to LSR ones (32.4%).

Despite the absence of statistically significant disparity in the pregnancy between the experimental groups (Table 4), it has been proven that the ram effect alters the sheep’s fertility, by acting as a synchronizer and inducing ovulation through the introduction of rams into sheep flocks. As posited by Martin et al. [56], this action instigates the secretion of gonadotropic hormones, promoting ovulation and increasing the probability of conception; they mention, in addition, that the pheromone stimulation emitted by rams in the environment activates females’ hormonal response, thereby synchronizing ovulation and improving fertility during copulation. However, the results obtained in this investigation, where some experimental groups reached an almost 100% pregnancy rate solely with the male effect (Table 4), do not coincide with those reported by Cadena-Villegas et al. [37], who found no difference in the number of pregnant females in Suffolk wool sheep, through hormonal protocols and bio-stimulation, where the pregnancy rate hovered at around 50%.

With regards to the number of embryos, it was the only response variable that showed a significant difference (*p* < 0.01) between experimental groups. The results obtained demonstrate that the HSR-E group exhibits a superior reproductive performance than its counterpart, showing, moreover, for the LSR-R, a higher level of performance in comparison with the HSR-R group. On the other hand, Martínez-Alfaro et al. [57] posit than the body condition exerts a significant influence on the induction of estrus and ovulation, increasing the follicle-stimulating hormone and luteinizing hormone production, and thus improving the survival of the embryo. In contrast, Cadena-Villegas et al. [37] reported that the male effect influenced prolificacy and fecundity in ewes treated with prostaglandin. Furthermore, the absence of statistically significant differences in morphological variables such as the body condition and live weight may be attributable to the uniformity in housing, feeding, and water conditions across all sheep. However, it is important to point out that HSR-E showed better conditions, as well as a higher number of embryos, in response to male stimuli. Conversely, the findings concerning the embryo size and ovary size were consistent with those reported by Fierro et al. [58], who obtained an average of 1.27 and 1.50 mm, in female subjects subjected to hormonal protocols. The statistical analysis indicated that there were no significant differences between morphometric variables between both social groups. It is, therefore, advisable to conduct a study with a larger number of males, in order to analyze whether a larger number of bucks might provide a better description of the effect that social rank may have had on the results.

## 5. Conclusions

The findings of this study demonstrate that the social rank significantly affects the frequency and expression of appetitive and consummatory interactions in sheep flocks in the northern region of Mexico. However, no statistically significant differences (*p* > 0.05) were found in reproductive variables such as estrus, ovulation, and pregnancy rates. Furthermore, the results obtained demonstrated that the males’ social rank does not influence the presence of corpus luteum and the total number of embryos. These are results that contrast with those obtained regarding the females, whose social rank exhibited a difference between both groups, a discrepancy that can be attributed to the fact that HSR females have better access to resources. In this regard, enhancing the reproductive variables has the capacity to contribute to the stabilization and/or improvement of the family’s income, who are reliant on sheep production. However, further research is required to comprehensively evaluate the underlying mechanisms of the observed results, and their potential implications for the management of sheep flocks. It is also evident that further study is required in conjunction with a larger-scale investigation involving a greater number of animals, in order to confirm the results for some variables.

## Figures and Tables

**Table 1:** Recorded confrontations during the competition test used to calculate the social rank index (high or low) and success index in Dorper rams (n = 33) and ewes (n = 59) in northern Mexico (25° N).

	Rams		Ewes	
	HSR ^1^	LSR ^2^	HSR	LSR
Rank index (%; n)	42.4 ^a^ (14/33)	57.6 ^a^ (19/33)	39.0 ^a^ (23/59)	61.0 ^a^ (36/59)
Events won (%; n)	68.8 ^a^ (55/80)	21.2 ^b^ (17/80)	87.8 ^a^ (129/147)	16.8 ^a^ (21/125)
Events lost (%; n)	31.2 ^a^ (25/80)	78.8 ^b^ (63/80)	12.2 ^a^ (18/147)	83.2 ^b^ (104/125)
Success index	0.69 ± 0.10 ^a^	0.21 ± 0.90 ^b^	0.88 ± 0.03 ^a^	0.17 ± 0.03 ^b^

^1^ HSR: high social rank; ^2^ LSR: low social rank. ^a, b^ Different superscript letters indicate statistical differences (*p* < 0.05).

**Table 2:** Mean ± standard deviation for morphometric variables in Dorper rams and ewes of high or low social rank in northern Mexico (25° N).

Variable	Rams		Ewes	
	HSR ^1^	LSR ^2^	HSR	LSR
Age (year)	3.9 ± 1.4 ^3^	3.1 ± 1.0	3.4 ± 0.5	3.0 ± 0.6
Live weight (kg)	61.5 ± 12.9	53.1 ± 10.2	40.4 ± 6.8	37.3 ± 5.7
Body condition (u)	3.2 ± 0.8	3.3 ± 1.0	2.6 ± 0.5	2.6 ± 0.5
Thoracic circumference (cm)	93.4 ± 6.4	90.4 ± 5.7	83.9 ± 7.5	81.3 ± 4.8
Wither height (cm)	68.0 ± 4.7	63.3 ± 3.4	64.0 ± 3.2	62.1 ± 3.9
Presence of horns	2/14 (14%)	4/19 (21%)		
Testicular circumference (cm)	31.1 ± 2.7	31.3 ± 2.6		

^1^ HSR: high social rank; ^2^ LSR: low social rank. ^3^ No differences were observed (*p* > 0.05) between the means of both ranges, of the same sex for the same variable.

**Table 3:** Mean ± standard deviation for the number of socio-sexual interactions in Dorper rams and ewes of high or low social rank in northern Mexico (25° N).

Interaction (n)	Experimental Group			
	HSR-R ^1^ × HSR-E ^2^	HSR-R × LSR-E ^3^	LSR-R ^4^ × HSR-E	LSR-R × LSR-E
Sniffing	42.0 ± 10.0 ^a^	62.6 ± 4.6 ^b^	31.3 ± 5.6 ^a^	24.9 ± 3.4 ^b^
Kicking	6.3 ± 1.6 ^a^	8.0 ± 1.8 ^b^	1.6 ± 0.7 ^a^	0.9 ± 0.2 ^b^
Flehmen	13.5 ± 4.4 ^a^	14.2 ± 1.2 ^b^	9.6 ± 1.3 ^a^	4.4 ± 1.0 ^b^
Knocking	5.0 ± 1.0 ^a^	11.3 ± 1.4 ^b^	0.9 ± 0.4 ^a^	1.7 ± 0.3 ^b^
Mounting	2.5 ± 0.8 ^a^	1.3 ± 0.2 ^a^	0.3 ± 0.1 ^a^	0.6 ± 0.1 ^b^
Mounting with penis unsheathed	1.5 ± 0.3 ^a^	2.2 ± 0.2 ^b^	0.4 ± 0.1 ^a^	0.3 ± 0.1 ^b^
Vocalizations	15.0 ± 2.5 ^a^	25.7 ± 1.1 ^b^	1.6 ± 0.2 ^a^	3.7 ± 0.8 ^b^

^1^ HSR-R: high-social-rank rams; ^2^ HSR-E: high-social-rank ewes; ^3^ LSR-E: low-social-rank ewes; ^4^ LSR-R: low-social-rank rams. ^a, b^ Different superscript letters indicate differences (*p* < 0.05) between the means of the interaction of males of the same rank with females of both ranks.

**Table 4:** Mean ± standard deviation for reproductive variables in Dorper ewes of high or low social rank exposed to Dorper rams of high or low social rank in northern Mexico (25° N).

Variable	Experimental Group (EG)				Ewes		Rams	
	HSR-R ^1^ × HSR-E ^2^	HSR-R × LSR-E ^3^	LSR-R ^4^ × HSR-E	LSR-R × LSR-E	HSR	LSR	EE ^5^ HSR-R	EE LSR-R
ER ^6^	23/23 (100) ^a^	28/36 (77.7) ^a^	21/23 (91.3) ^a^	36/36 (100) ^a^	91.3 ^a^	88.8 ^a^	86.2 ^a^	93.3 ^a^
OR ^7^	21/23 (91.3) ^a^	30/36 (83.3) ^a^	23/23 (100) ^a^	30/36 (83.3) ^a^	95.6 ^a^	83.3 ^a^	86.2 ^a^	90.0 ^a^
CL ^8^	1.3 ± 0.4 ^a^	1.0 ± 0.2 ^a^	1.3 ± 0.5 ^a^	0.8 ± 0.2 ^a^	1.3 ± 0.4 ^a^	0.9 ± 0.3 ^b^	1.1 ± 0.3 ^a^	1.0 ± 0.2 ^a^
LO ^9^	11.3 ± 1.6 ^a^	8.5 ± 2.4 ^a^	10.1 ± 2.3 ^a^	10.2 ± 2.4 ^a^	10.7 ± 2.3 ^a^	9.6 ± 1.5 ^a^	9.1 ± 2.4 ^a^	11.0 ± 2.2 ^a^
RO ^10^	2.2 ± 1.3 ^a^	2.3 ± 0.4 ^a^	3.8 ± 1.4 ^a^	0.5 ± 0.2 ^a^	3.0 ± 0.4 ^a^	1.3 ± 0.3 ^a^	2.8 ± 0.4 ^a^	1.2 ± 0.3 ^a^
PR ^11^	19/23 (82.6) ^a^	36/36 (100) ^a^	23/23 (100) ^a^	30/36 (83.3) ^a^	90.9 ^a^	91.6 ^a^	93.1 ^a^	89.6 ^a^
EM ^12^	1.3 ± 0.3 ^ab^	1.3 ± 0.4 ^ab^	1.8 ± 0.2 ^a^	0.9 ± 0.3 ^b^	1.5 ± 0.6 ^a^	1.1 ± 0.2 ^b^	1.3 ± 0.5 ^a^	1.3 ± 0.4 ^a^

^1^ HSR-R: high-social-rank rams; ^2^ HSR-E: high-social-rank ewes; ^3^ LSR-E: low-social-rank ewes; ^4^ LSR-R: low-social-rank rams; ^5^ EE: ewes exposed; ^6^ ER: estrous rate (%); ^7^ OR: ovulation rate (%); ^8^ CL: corpus luteum (n); ^9^ LO: left ovary (mm); ^10^ RO: right ovary (mm); ^11^ PR: pregnancy rate (%); ^12^ EM: embryos (n). ^a, b^ Different superscript letters indicate differences (*p* < 0.05) between the means.

## Data Availability

None of the data were deposited in an official repository, yet information can be made available upon request.

## Abbreviations

The following abbreviations are used in this manuscript:

HSR	High Social Rank
LSR	Low Social Rank
SI	Success Index
HSR-R	High-Social-Rank Rams
HSR-E	High-Social-Rank Ewes
LSR-R	Low-Social-Rank Rams
LSR-E	Low-Social-Rank Ewes
